# Future of microbial polyesters

**DOI:** 10.1186/1475-2859-12-54

**Published:** 2013-05-28

**Authors:** Gi Na Lee, Jonguk Na

**Affiliations:** 1 Korean Minjok Leadership Academy, 600 Bongwha-ro, Anheung-myeon, Hoengseong-gun, Gangwon-do 225-823, Republic of Korea

**Keywords:** Polyester, Polyhydroxyalkanoate, Metabolic engineering

## Abstract

Numerous microorganisms accumulate polyesters classified as polyhydroxyalkanoates (PHAs) as carbon and energy storage material when the growth condition is unfavorable in the presence of excess carbon source. Natural PHAs typically consist of various (*R*)-hydroxycarboxylic acids, and exhibit different material properties depending on the monomer composition. Such diversity comes from different metabolic pathways operating in the cell, and thus generating different monomers. Even more diverse PHAs can be produced by metabolically engineered microorganisms, which leads to the biosynthesis of non-natural polyesters containing lactate as a monomer. In order to make PHAs as useful polymers in our daily life, their production cost should be significantly lowered and material properties should be compatible with those produced by petrochemical industries. Metabolic engineering can address these issues by developing microbial strains capable of producing PHAs of desired material properties with high productivity and yield from inexpensive carbon sources. This commentary aims at peeking into the future of PHAs, focusing on the possible metabolic engineering strategies to be taken to achieve these goals.

## Introduction

Plastics we use every day are made from fossil oil and natural gas through petroleum refinery process. They are light, durable, inexpensive, easy to make various articles, and long lasting, which made them so popular in manufacturing various articles from simple containers and fibers to engineering plastics. As fossil resources will ultimately be depleted, it is necessary to develop alternative processes for the production of future plastics. Climate change and other environmental problems are also warning us that more sustainable ways of manufacturing plastics need to be developed.

Polyhydroxyalkanoates (PHAs) are polyesters synthesized by numerous microorganisms [1,2]. Different microorganisms were found to accumulate various PHAs comprises (*R*)-hydroxycarboxylic acids, having a carboxyl group at the end and a hydroxyl group at 3-, 4-, 5-, or 6-position; to date, more than 150 kinds of hydroxycarboxylic acids have been found as the monomers of PHAs [3]. For convenience reasons, researchers classified PHAs into two groups depending on the total number of carbons in the monomer: short-chain-length (SCL)-PHAs having 3 to 5 carbon atoms and medium-chain-length (MCL)-PHAs having 6 to 14 carbon atoms [2,4]. SCL-PHAs show thermoplastic material properties similar to polypropylene, while MCL-PHAs possess elastic material properties similar to rubber. Interestingly, some microorganisms synthesize PHAs having both SCL and MCL-monomers. Such SCL-MCL-PHAs exhibit material properties similar to low density polyethylene [4,5]. Recently, some PHA homopolymers and block copolymers have been produced by metabolically engineered bacteria, further extending the PHA diversity [6-9]. Thus, it is possible to produce diverse family of PHAs possessing material properties similar to many polymers we currently use.

Even though PHAs are such great materials, they are not being used widely. Two major reasons are relatively high cost of production and inferior material properties compared to the petroleum-based plastics. In this commentary, we suggest some strategies that can be taken to address these problems.

### Lowering the production costs of PHAs

PHAs can be produced by fermentation of microorganisms, whether they are natural isolates or engineered ones. Factors affecting the overall cost of PHA production include the cost of raw materials including carbon source, the PHA yield on carbon source, PHA productivity and recovery and other downstream costs [10]. Thus, the solution to the high cost problem can be theoretically solved by using inexpensive carbon source, achieving high PHA yield and productivity and low downstream costs [2,10]. Interestingly, these are interlinked and can be solved by metabolic engineering.

First, the cheap raw materials can be chosen depending on the region where PHA production plant is in operation. Raw materials that are abundant in that region of production site can often be, but not always though, inexpensive substrates for PHA production; for example, sucrose (sugar cane) is one of the best substrates in Brazil and Queensland, Australia. What will happen if the microorganism capable of producing desired PHA cannot utilize the most inexpensive carbon source? Let’s assume that recombinant *Escherichia coli* K12 strain was developed by metabolic engineering to produce SCL-MCL PHA. If sucrose is the carbon source to be used, two options can be considered to address the inability of *E. coli* K12 strain to utilize sucrose. First, the sucrose utilization pathways can be introduced into the strain producing PHAs. Second, the strain already constructed can be discarded and a new sucrose-utilizing strain can be engineered to biosynthesize PHAs. If the lignocellulosic hydrolysate is the preferred carbon substrate, the strain should be developed to utilize both glucose and xylose equally efficiently, while making it tolerant to many toxic chemicals present therein.

For the production of PHA with high yield and productivity, more serious metabolic engineering needs to be performed. In the case of poly(3-hydroxybutyrate), (P3HB), the main metabolite precursor is acetyl-CoA as it is condensed to form acetoacetyl-CoA followed by reduction and polymerization [2,4]. Since byproducts formation needs to be minimized to increase the PHA yield, metabolic engineering is performed to eliminate the formation of acetic acid, lactic acid, formic acid and others that are produced during the cultivation. One interesting point is the generation of carbon dioxide during the conversion of pyruvate to acetyl-CoA. In the case of P3HB, for example, the theoretical maximum carbon yield of P3HB on glucose is 66.7%. Although the strategy for utilizing the evolved carbon dioxide is not clear at this moment, it will be desirable to fix carbon dioxide through a new enzyme and pathway, which can consequently be converted to acetyl-CoA.

PHA production in industrial-scale fermentation will be performed in a fed-batch mode to increase the productivity and concentration, as in the cases for many other industrial fermentation processes [2]. The PHA productivity can be maximized by the optimization of cell mass formation and the specific PHA productivity at the same time. It is particularly important to closely examine the relationship between cell growth and PHA formation because PHA is an intracellular product which physically occupies the cytosol. Because of this, accumulation of large amounts of PHA inhibits cell growth and often negatively affects normal cell metabolism [2,4]. If the developed strain synthesizes PHA too efficiently (fast), cells will be full of PHA granules too early, resulting in lower overall productivity. If the developed strain synthesizes PHA too slowly, much carbon source will be wasted to cell growth with less PHA accumulation inside the cell. Optimization of cell growth and PHA biosynthesis rates will vary depending on the microorganism employed, and thus needs to be determined by experiments. Therefore, metabolic engineering of microorganism should be performed in the context of optimal fermentation.

Since PHAs are accumulated inside the cell, cells need to be disrupted to recover PHAs after fermentation [2,4]. Extraction of PHAs with solvents such as chloroform does not make much sense considering that PHAs are environmentally friendly polymer while chloroform is one of the environmentally worst solvents. It is interesting to note that PHAs could be efficiently purified by simple treatment with mild alkaline solution from recombinant *E. coli* accumulating a large amount of PHAs (e.g., greater than 85% of dry cell weight as PHAs) [11]. This was possible due to that cells became extremely fragile after the accumulation of such large amounts of polymer inside the cell. Thus, it is clear that metabolic engineering of strains and fermentation need to be performed to allow maximum accumulation of PHA granules inside the cell at the end of fermentation; again, too early accumulation of that much PHA will result in lower overall productivity. On the other hand, metabolic engineering approach can be taken to construct induced lysis for the PHA producing cells to release its PHA granules, allowing cost saving for PHA purification. Also, it should be mentioned that PHAs can be produced in plants directly from carbon dioxide and sunlight as having been studied for more than a decade [12]. Although the PHA contents achieved in plants to date are rather low, it will be interesting to see how much PHA accumulation can be increased as more metabolic engineering effort is exerted.

In summary, the production cost of PHA can be lowered by using inexpensive carbon substrates, developing a strain that is capable of producing PHA at the optimal rate so that the high PHA content and high overall productivity can be achieved at the end of fed-batch culture, and establishing a simple yet environmentally friendly recovery processes of low operating costs. Towards these goals, it is important to develop the improved strains by metabolic engineering and fermentation-recovery processes in an integrated manner [10,13].

### Improving the material properties of PHAs

As mentioned above, PHAs exhibit a wide range of material properties depending on the monomer units. In this paper, it is not intended to review the state-of-the-art of various application possibilities based on such diverse material properties of PHAs. Readers are encouraged to consult several excellent papers on this topic [1,12,13]. Here, instead, we would like to suggest the strategies for further improving the material properties of PHAs. Demonstration of great LDPE-like properties by producing SCL-MCL-copolymer PHAs was a good example of how the material properties of PHAs can be improved. More recently, even the non-natural polyesters like polylactic acid (PLA) and lactate-containing PHAs could be produced by metabolic engineering [14,15]. Also, it has become possible to design and synthesize PHA with defined blocks for better properties [6-9]. It was essential to create two enzymes: one that converts lactate to lactyl-CoA and the other polymerizes lactyl-CoA into the growing chain of the polymer [14,15]. Thus, it is clear that metabolic engineering strategies that allow generation of CoA-charged hydroxycarboxylic monomers together with protein engineering of PHA synthases will allow biosynthesis of even more diverse family of PHAs. With our ability to engineer PHA synthases to have much broad substrate specificities, the PHA biosynthesis system can become a truly versatile platform for the manufacture of diverse and even non-natural polyesters. Among these newly produced PHAs, we will be able to find polymers exhibiting similar to or even better material properties than the petroleum-derived plastics currently used.

What lies in the future? If we look at the broad spectrum of plastics in use nowadays, there is clearly an important missing family of polyesters we want to produce through biotechnology: aromatic polyester. For example, polyethylene terephthalate (PET) is widely used in synthetic fibers and beverage and liquid containers. Its monomer is synthesized by the esterification of an aromatic chemical terephthalic acid and ethylene glycol. Although PHAs consisted of aromatic monomers have been biosynthesized, the aromatic rings were always on the side chains rather than the main polymer chain. Thus, it will be interesting to see if such aromatic monomers like terephthalic acid can be incorporated into PHAs to make polymers similar to PET. One possible way to achieve this is the development of a new metabolic pathway that can generate hydroxy-terephthalyl-CoA *in vivo* and an evolved PHA synthase that can accept it as a monomer during the polymerization. Considering the unprecedentedly rapid advances in metabolic engineering, synthetic biology and evolutionary engineering [16], we are optimistic that such new polymers can be biosynthesized at low costs from inexpensive substrates in the near future.

### Future perspectives

**Figure 1:**
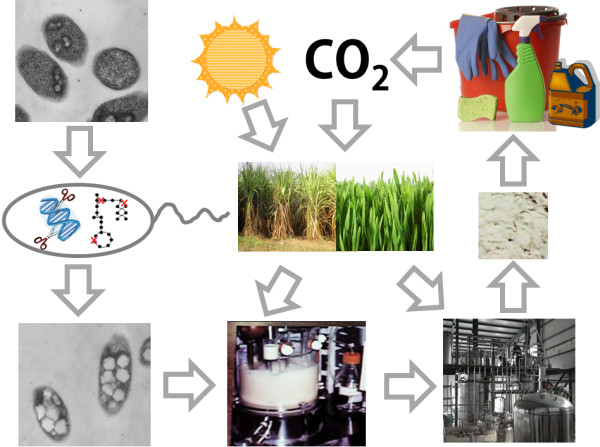
**Production of microbial polyesters by fermentation and its complete carbon cycle.** Microorganisms isolated from nature are metabolically engineered to accumulate a large amount of plastics with high yield and specific productivity (left). Biomass obtained from carbon dioxide and sunlight is converted to fermentable sugars, and used as a substrate for lab-scale fermentation for the examination of the performance and further strain optimization. Once a high performance microorganism is developed, industrial-scale fermentation, after process optimization to give the highest possible yield and productivity, is performed to produce large amounts of plastics. After fermentation, polymers inside cells are purified and used to make articles we use everyday. When they are disposed after use, they will be degraded to carbon dioxide (and methane under anaerobic condition). Thus, the carbon cycle becomes closed, providing environmentally friendly sustainable way of producing plastics without using fossil oil.

Plastics are one of the greatest inventions of humans. It is difficult to think our world without plastics. However, will we be able to produce plastics in year 2500 in the same way as we do through petroleum refineries now? Probably not, as all the fossil resources will be depleted by then. Thus, it is essential for us to develop more sustainable processes for the production of plastics. Bio-based production of plastics from renewable biomass, and maybe directly from carbon dioxide in the future, can be realized in general in two ways. One is the bio-based production of monomers, followed by chemical polymerization process we use nowadays. Although this topic was not covered in this commentary, it is indeed a great strategy for more sustainable production of plastics as nicely reviewed previously [13,16,17]. The other is the fermentative production of plastics by metabolically engineered microorganisms as discussed in this paper. Chemical companies have not yet been willing to produce plastics by bio-based processes due to the high production cost and inferior material properties; although it seems to be slowly changing now [1]. As discussed earlier, metabolic engineering is allowing us to develop high performance microorganisms capable of producing chemicals and materials cost effectively. When combined with bioprocess development, many economically feasible bioprocesses for the production of plastics will be realized. Once the process becomes economical, the bio-based production of plastics indeed forms a complete, environment-friendly carbon cycle (Figure 1). As readers will mostly agree, we are responsible for the future of humankind and our environment. After all, *“…We have not inherited the land from our fathers, we have borrowed it from our children …”*[18].

## Competing interests

The authors declare no competing financial interest.

## Authors’ contributions

GNL and JN wrote the commentary together, and read and approved the final version of the commentary.
